# A Rare Case of Streptococcal Toxic Shock Syndrome Associated With Pneumonia

**DOI:** 10.7759/cureus.80620

**Published:** 2025-03-15

**Authors:** Suguru Hasegawa

**Affiliations:** 1 Intensive Care Medicine, Toyama Prefectural Central Hospital, Toyama, JPN

**Keywords:** clindamycin, community-acquired pneumonia, group a streptococcus, shock, streptococcal toxic shock syndrome

## Abstract

Streptococcal toxic shock syndrome (STSS) is a rapidly progressing and life-threatening illness caused by group A Streptococcus (GAS), typically associated with invasive infections such as necrotizing fasciitis and characterized by high mortality rates. Although GAS is an uncommon cause of community-acquired pneumonia (CAP), it can lead to severe illness in both healthy individuals and those with comorbidities. We report a case of STSS with pneumonia in a previously healthy 69-year-old female who presented with severe respiratory distress and shock without skin lesions. Despite the rapid progression typical of STSS, the patient showed remarkable clinical improvement with tazobactam/piperacillin and azithromycin, without the administration of clindamycin. This study challenges the conventional approach that emphasizes clindamycin due to its antitoxin effects, highlighting the importance of individualized therapeutic decisions. This case underscores the necessity of considering STSS in the differential diagnosis of severe respiratory infections in adults, even in the absence of skin manifestations. Further research is needed to clarify the role of antitoxin therapies and to establish evidence-based guidelines for managing STSS, particularly in cases presenting with pneumonia without skin involvement.

## Introduction

Streptococcal toxic shock syndrome (STSS) is a severe and rapidly progressing illness caused by group A Streptococcus (GAS) pyogenes, characterized by the sudden onset of fever, shock, and multiorgan failure. Despite advances in medical care, STSS continues to be associated with high mortality rates. STSS is often linked to invasive infections such as necrotizing fasciitis and typically begins with fever, pain, and skin manifestations, rapidly progressing to shock and multiorgan dysfunction [1]. Treatment involves aggressive fluid resuscitation, targeted antibiotic therapy (such as penicillin or beta-lactams), and often surgical debridement in cases of associated necrotizing fasciitis [2]. Clindamycin has emerged as a key component of standard treatment for severe cases due to its antitoxin effects [3].

Although GAS is an uncommon cause of community-acquired pneumonia (CAP), it can affect both healthy individuals and those with comorbidities, leading to high mortality rates [4]. Early recognition and prompt initiation of appropriate treatment, including antitoxin therapy, are crucial for improving outcomes in STSS associated with pneumonia [5]. We report a case of STSS associated with CAP in which the patient presented in a severe state of shock upon arrival at our hospital but was successfully treated and discharged home without the administration of clindamycin.

## Case presentation

The patient is a 69-year-old female who had been caring for her grandchild with a cold two days prior to the onset of symptoms. She developed a fever on the evening of that day. From midday the following day, she gradually experienced increasing difficulty in moving her body, and on the day of emergency transportation, she became completely immobile, prompting an emergency call.

Her medical history was significant for hypertension, managed with a single antihypertensive medication. She had no other significant medical history, no known allergies, and no history of smoking or alcohol consumption. Her activities of daily living were completely independent before the onset of symptoms.

Upon arrival at our hospital, her vital signs were as follows: blood pressure of 136/76 mmHg, heart rate of 127 beats per minute, body temperature of 39.9°C, and respiratory rate of 24 breaths per minute. Despite receiving 10 L of oxygen via a reservoir mask, her SpO_2_ was 83%, indicating severe hypoxemia. Consequently, endotracheal intubation was performed in the emergency room. The initial arterial blood gas analysis performed after endotracheal intubation revealed a PaO_2_ of only 58.3 mmHg, despite mechanical ventilation with an inspired oxygen concentration of 80%.

**Figure 1:**
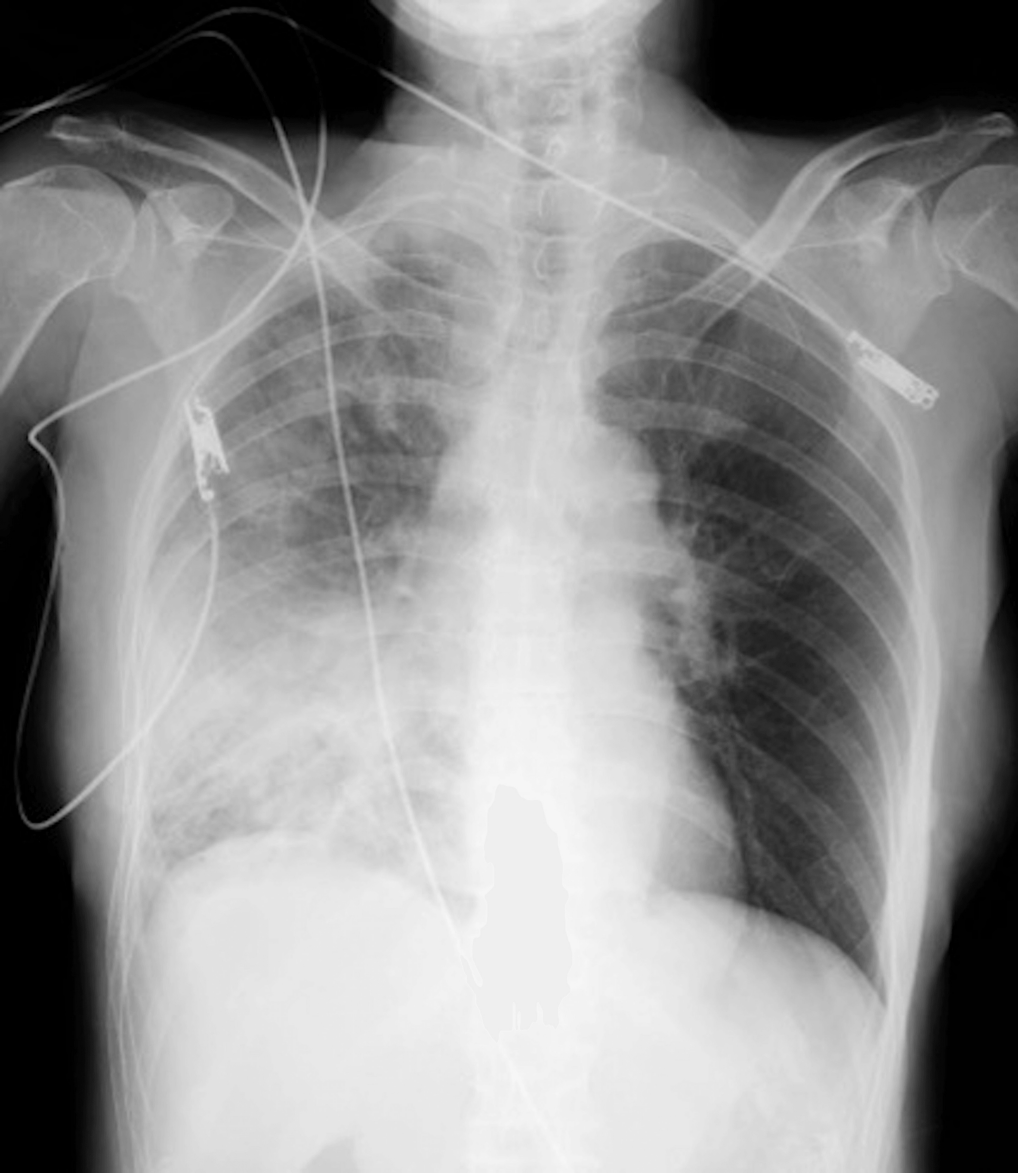
The chest radiograph obtained at the presentation. The imaging showed widespread decreased translucency in the right lung field, predominantly in the lower lung area, suggesting findings consistent with pneumonia.

**Figure 2:**
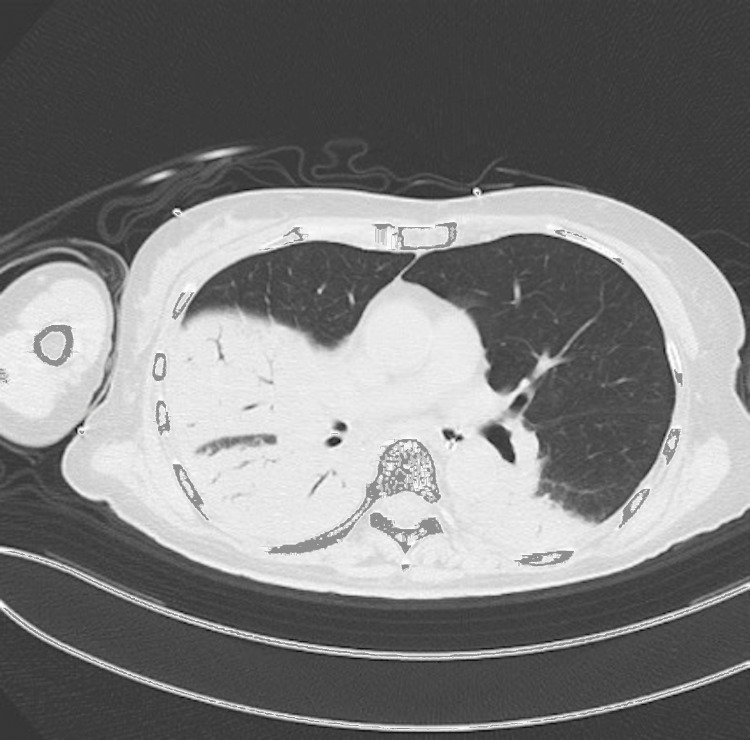
Chest computed tomography on admission. The imaging revealed extensive dense consolidations in the right middle and lower lobes, consistent with a diagnosis of pneumonia.

Thoracic computed tomography (CT) revealed pneumonia in the right middle and lower lobe (Figures 1, 2). Blood tests revealed a white blood cell count of 6,200/μL, which was within the normal reference range; however, C-reactive protein (CRP) was markedly elevated at 38.07 mg/dL. Additionally, lactate dehydrogenase (LD) and creatine kinase (CK) levels were elevated, accompanied by mild elevations in liver enzymes and mild renal impairment (Table 1). Sputum Gram staining revealed a Geckler classification of 5, with 3+ Gram-positive cocci, a small amount of Gram-negative cocci, and 3+ white blood cells. Additionally, a few instances of white blood cells phagocytosing Gram-positive cocci were observed.

**Table 1:** Findings of blood tests at the time of admission. A white blood cell count was 6,200/μL, which falls within the normal reference range; however, C-reactive protein was markedly elevated at 38.07 mg/dL. Additionally, lactate dehydrogenase and creatine kinase levels were elevated, accompanied by mild elevations in liver enzymes and mild renal impairment.

Parameters	Patient values	Reference range
WBC	6,200/μL	3,300-8,600/μL
Hb	11.8 g/dL	11.6-14.8 g/dL
MCV	91.8 fL	83.6-98.2 fL
MCH	31.2 pg	27.5-33.2 pg
MCHC	34 g/dL	31.7-35.3 g/dL
PLT	168,000/μL	158,000-348,000/μL
PT-INR	1.28	0.85-1.15
APTT	35.8 s	24-39 s
FDP	23.9 μg/mL	<5.0 μg/mL
TP	6.1 g/dL	6.6-8.1 g/dL
ALB	2.7 g/dL	4.1-5.1 7 g/dL
AST	40 U/L	13-30 U/L
ALT	35 U/L	7-30 U/L
ALP	39U/L	38-113 U/L
LD	329 U/L	124-222 U/L
CK	811 U/L	41-153 U/L
r-GT	20 U/L	9-50 U/L
AMY	34 U/L	44-132 U/L
T-Bil	0.6 mg/dL	0.4-1.5 mg/dL
BUN	41 mg/dL	8-20 mg/dL
CRE	1.41 mg/dL	0.46-0.70 mg/dL
UA	5.8 mg/dL	2.6-7.0 mg/dL
Na	132 mmol/L	138-145 mg/dL
K	3.2 mmol/L	3.6-4.8 mmol/L
Cl	98 mmol/L	101-108 mmol/L
CRP	38.07 mg/dL	0.0-0.14 mg/dL

Various cultures were promptly obtained, and treatment was initiated with tazobactam/piperacillin, azithromycin, and corticosteroids. The patient also exhibited severe dehydration and hemodynamic instability, which were managed with fluid resuscitation, norepinephrine, vasopressin, and epinephrine. Her hemodynamic status rapidly stabilized, allowing the discontinuation of all vasopressors by hospital day four.

By hospital day three, group A Streptococcus (GAS) had been identified from the initial blood cultures and sputum culture. However, due to the patient’s improving overall condition, clindamycin administration was deemed unnecessary. Her arterial oxygen levels showed a daily trend of improvement. On the morning of hospital day five, arterial blood gas analysis revealed a PaO_2_ of 63.8 mmHg while the patient was on mechanical ventilation with an inspired oxygen concentration of 30%. Based on this improvement, she was successfully extubated later that day. She was transferred to a general ward on hospital day seven.

By hospital day 17, oxygen therapy was no longer required. Following improvement in activities of daily living through rehabilitation, she was discharged home on hospital day 28. A chest x-ray prior to discharge showed that the pneumonia image had disappeared.

## Discussion

STSS is a severe complication of invasive GAS infections, characterized by the rapid onset of shock and multiple organ failure. It is associated with high mortality rates due to its rapid progression from onset to death. Effective management of STSS requires early recognition, intensive organ support, rapid identification of infection sources, and surgical intervention when necessary [2].

In adults, STSS typically manifests following skin or soft tissue infections, such as cellulitis or necrotizing fasciitis. Conversely, pediatric cases often arise from respiratory tract infections without accompanying skin lesions. This distinction underscores the necessity for clinicians to maintain a high index of suspicion for STSS in pediatric patients presenting with severe respiratory symptoms, even in the absence of cutaneous manifestations [6].

While the association between pediatric respiratory infections and STSS is well-documented, the occurrence of STSS originating from respiratory infections without skin manifestations in adults is less commonly reported. However, recent epidemiological data indicate an increasing trend in STSS cases among adults, with a notable rise in infections linked to respiratory pathogens. For instance, the National Institute of Infectious Diseases in Japan reported a significant increase in STSS cases in 2023, with a substantial proportion associated with respiratory tract infections.

The pathophysiological mechanisms underlying STSS in the context of respiratory infections involve the invasion of streptococci into normally sterile sites, leading to a systemic inflammatory response. In adults, factors such as immunosenescence, comorbidities, and potential delays in seeking medical attention may contribute to the severity and atypical presentations of STSS. Therefore, it is imperative for healthcare providers to consider STSS in the differential diagnosis when encountering adult patients with severe respiratory infections, even in the absence of skin lesions. Early recognition and prompt initiation of appropriate antimicrobial therapy, along with supportive care, are crucial in managing STSS. Given the potential for rapid deterioration, clinicians should maintain a high degree of vigilance for this condition across all age groups and clinical presentations.

In the present case, the possibility of STSS was not initially considered upon admission. Based on imaging findings and Gram staining results, treatment was commenced with tazobactam/piperacillin and azithromycin to cover for lobar pneumonia and atypical pneumonia, without administering clindamycin. Despite this, the patient's clinical course was remarkably favorable. This raises the following question: had the potential for STSS been considered from the outset, should clindamycin have been administered?

Treatment of STSS typically includes clindamycin to inhibit toxin production, but emerging resistance in *Staphylococcus aureus* and *S. pyogenes *has raised questions about its efficacy [7]. However, a Swedish study found that both clindamycin and intravenous immunoglobulin (IVIG) therapy significantly improved survival in STSS patients [8]. Conversely, a retrospective study in China reported high mortality rates in pediatric STSS cases, with most isolates resistant to clindamycin and erythromycin [9]. Despite concerns about clindamycin resistance, one study suggested that withholding anti-toxin agents or administering clindamycin to resistant organisms did not adversely affect patient outcomes [5].

These findings suggest that while clindamycin is recommended for STSS, individual patient factors, and clinical judgment play crucial roles in therapeutic decisions. This case demonstrates that favorable outcomes can be achieved even without clindamycin, challenging the conventional approach. Further research is warranted to clarify the specific circumstances under which clindamycin administration is essential in the management of STSS, particularly in cases involving respiratory infections without skin manifestations.

## Conclusions

In this study, a previously healthy adult developed STSS accompanied by pneumonia without any associated skin lesions. Despite the absence of clindamycin administration, the patient showed a favorable clinical course. When managing adult patients with severe respiratory infections, it is crucial to consider STSS in the differential diagnosis. Further research is needed to establish optimal treatment strategies for STSS, including the appropriate selection of antimicrobial agents.

## References

[1] Agerson AN, Wilkins EG (2005). Streptococcal toxic shock syndrome after breast reconstruction. Ann Plast Surg.

[2] Schmitz M, Roux X, Huttner B, Pugin J (2018). Streptococcal toxic shock syndrome in the intensive care unit. Ann Intensive Care.

[3] Stevens DL (2000). Streptococcal toxic shock syndrome associated with necrotizing fasciitis. Annu Rev Med.

[4] Tamayo E, Montes M, Vicente D, Pérez-Trallero E (2016). Streptococcus pyogenes pneumonia in adults: clinical presentation and molecular characterization of isolates 2006-2015. PLoS One.

[5] Makino H, Iwata S, Kouda T, Kato T, Kajiwara K, Hamaguchi N (2017). Pneumonia and streptococcal toxic shock syndrome due to group A streptococci: a case report. [Article in Japanese]. Kansenshogaku Zasshi.

[6] Chiang MC, Jaing TH, Wu CT, Hsia SH, Chiu CH (2005). Streptococcal toxic shock syndrome in children without skin and soft tissue infection: report of four cases. Acta Paediatr.

[7] Perez CS, Nuibe AM (2019). 472. Rethinking the role of clindamycin for toxin-mediated illnesses. Open Forum Infect Dis.

[8] Linnér A, Darenberg J, Sjölin J, Henriques-Normark B, Norrby-Teglund A (2014). Clinical efficacy of polyspecific intravenous immunoglobulin therapy in patients with streptococcal toxic shock syndrome: a comparative observational study. Clin Infect Dis.

[9] Hua CZ, Yu H, Yang LH (2018). Streptococcal toxic shock syndrome caused by Streptococcus pyogenes: a retrospective study of 15 pediatric cases. [Article in Chinese]. Zhonghua Er Ke Za Zhi.

